# Attitude towards COVID-19 vaccination among healthcare workers: a cross sectional study from Egypt

**DOI:** 10.1186/s12913-022-08751-3

**Published:** 2022-11-16

**Authors:** Samar Tharwat, Dalia Kamal Nassar, Mohammed Kamal Nassar, Ahmed Mohammed Saad, Fatma Hamdy

**Affiliations:** 1 Rheumatology & Immunology Unit, Department of Internal Medicine, Faculty of Medicine, Mansoura University, El Gomhouria St, Mansoura, Dakahlia Governorate Egypt; 2 Medical Microbiology and Immunology Department, Faculty of Medicine, Mansoura University, Mansoura, Egypt; 3 Mansoura Nephrology & Dialysis Unit (MNDU), Department of Internal Medicine, Faculty of Medicine, Mansoura University, Mansoura, Egypt; 4 Faculty of Medicine, Mansoura University, Mansoura, Egypt

**Keywords:** COVID-19, Vaccine acceptance, Vaccine hesitancy, Health care workers, Egypt

## Abstract

**Background:**

Herd immunity is necessary to contain the coronavirus disease 2019 (COVID-19) pandemic. Vaccination is the fastest and safest pandemic control strategy. Healthcare workers (HCWs) are essential in providing vaccination information. The aim of this study was to assess intent to be vaccinated against COVID-19 among HCWs in Egypt and to determine the factors that may influence their decision.

**Methods:**

A questionnaire-based cross-sectional study was conducted among HCWs who care for patients in several hospitals in Delta region, Egypt. The questionnaire included sociodemographic, clinical, and occupational data, intention to receive the COVID-19 vaccine, and beliefs and attitudes towards COVID-19 and its vaccination.

**Results:**

The study included 455 HCWs with a mean age of 36.55 years (SD = 10.31) and 80% were females. The acceptance rate for the COVID-19 vaccine was 70.5%, while hesitancy and resistancy were both 17.6 and 11.9% respectively. About one-third (33.4%) of the subjects had previously contracted COVID-19. Most participants believed that they had a higher risk of contracting COVID-19 (71.6%). More than 64% believed they were at risk for vaccination side effects. Fear of infection and being at high risk of infection were the main drivers for COVID-19 vaccination, while the major barriers were waiting for additional experience with these new vaccines and having doubts about the vaccines’ efficacy.

**Conclusions:**

The acceptance of the COVID-19 vaccination among HCWs is very high. This crucial group needs to be the focus of educational initiatives and campaigns designed to increase public awareness of the safety and effectiveness of COVID-19 vaccination.

## Introduction

Globally, the severe acute respiratory syndrome coronavirus 2 (SARS-CoV-2) caused coronavirus disease 2019 (COVID-19) pandemic poses a serious threat [1], instigating a pandemic affecting more than 185 countries [2]. The pandemic has crippled global economic activity, overloaded hospital systems, and induced panic among the general population [3].

Vaccination is one of the most important public health measures to stop the spread of certain infectious diseases and to lower their mortality rate [4]. According to the World Health Organization (WHO), vaccines prevented at least 10 million deaths between 2010 and 2015 [5]. To stop the COVID-19 pandemic, high vaccination rates are needed worldwide [6]. Since the emergence of this new corona virus, numerous organizations around the globe have conducted substantial research in an effort to create a vaccine that will protect people from this deadly new virus in a safe and efficient manner [7].

Globally, worries about vaccine hesitancy are rising, particularly in populous nations with poor literacy rates. The definition of vaccine hesitancy is “delay in accepting or refusing vaccinations notwithstanding the availability of vaccination services” [8].

Healthcare workers (HCWs) play a critical role in offering advice and recommendations to patients and the larger community about vaccination, including accurate information about the risks and advantages of the vaccine [9]. A Values Framework for the Allocation and Prioritization of COVID-19 Vaccination was released in September 2020 and recommends that governments give priority to HCWs, older people, and those with chronic diseases to receive the first doses of an approved COVID-19 vaccine [10].

Among the most trusted sources of information about vaccines and vaccination for the general public are the HCWs [11]. But not all HCWs enthusiastically accept or advocate COVID-19 vaccination. To enhance vaccination uptake, HCWs must be targeted with supportive communication [11]. However, there is little information about the beliefs and attitudes of Egyptian HCWs towards COVID-19 vaccination.

Thus, the aim of this study was to assess intent to be vaccinated against COVID-19 among HCWs in Egypt and to determine the factors that may influence their decision to delay or refuse to receive the vaccine.

## Materials and methods

### Study population

This questionnaire based cross-sectional study was conducted in the duration from August to September 2021. The main population of interest was HCWs who care for patients in several hospitals in Delta region, Egypt. Anyone involved in the delivery of healthcare services, including those who interact directly with patients and those who do not, was generically referred to as a HCW. Thus, a variety of healthcare positions were included by this term such as physicians, pharmacists, radiology, and laboratory technicians ...etc. The study was approved by the institutional review board of Mansoura University (Approval No: R.21.08.1398.R1) and was consistent with declaration of Helsinki 1995. All participants received comprehensive information regarding the study, and their written informed consent was obtained.

### Sample size and sampling procedure

Healthcare staff employed by the hospitals were asked to participate in the study. Data were gathered using convenience sampling. The appropriate sample size was determined using the RaoSoft® online sample size calculator. We assumed the population size (current HCWs in Egypt) to be 375 thousand as provided by the most recent report of The Central Agency for Public Mobilization and Statistics (CAPMAS) [12]. Based on 50% predicted response, 5% margin of error and 80% degree of precision 95% confidence level, the minimum sample size was 385 participants.

### Survey

The questionnaire was written in English and a bilingual study author translated it into Arabic. Following editing and review, five medical staff members examined the questionnaire design, content, wording, and simplicity of completion as part of a pilot study that validated the questionnaire. Based on this, two new items were added, four were removed, and five were reworded. Then, a preliminary questionnaire was developed and pilot-tested with a small sample of HCWs (*n* = 22). The internal consistency of the questionnaire was determined using Cronbach’s alpha coefficient. The reliability coefficient was 0.85, indicating that the internal consistency was good. The data of those who participated in the pilot study were subsequently omitted from the statistical analysis of the study. The questions were designed to be as simple and closed-ended as feasible except for the assessment of opinions towards conventional vaccines.

### Survey administration

Interviews served as the basis for the study. It was intended to be completed in between 10 and 15 min. Early in the day, the interviewer visited the hospitals and spoke with as much HCWs as possible. All healthcare staff who volunteered to participate were interviewed during the interviewer’s visits to the hospitals. Face-to-face structured interviews were conducted by a single interviewer with each participant. All researchers contributed to the interviews with the HCWs. This mode of questioning makes it feasible to study complex issues than is possible in self-administered modes of questioning as the interviewer can provide more detailed explanations of the questions. Participants’ anonymity and secrecy were guaranteed by not requesting any personal information.

### Questionnaire and variables gathered

To adapt the questionnaire to the setting of our study, we included the significant items that were found based on previous literature findings [13–16]. The variables evaluated include:

#### Sociodemographic and clinical data

The data collected included 8 questions about gender, age, marital status, residence, smoking habit, socioeconomic status, and associated comorbidities.

#### Occupational data

The participants were asked 4 questions that covered their occupation and occupation settings whether offices, laboratories, inpatient wards, intensive care units or others. Information about dealing with patients or interacting with them was also recorded along with the frequency of contact with COVID-19 patients in the workplace.

#### Clinical data of SARS-CoV-2 infection

Information was collected related to history and severity of *SARS*-*CoV*-*2* infection among participants and their household or close friends. This section included 6 questions.

#### Perception, beliefs and attitudes towards COVID-19 and vaccination

Multiple questions about the perception of COVID-19 were also included. To determine the beliefs and attitudes towards conventional vaccination, the participants were asked to score their perception of efficacy, security, usefulness, and estimated knowledge of conventional vaccination in general where 0 was the lowest score and 10 was the highest score [17].

Additionally, 11 questions about attitudes and convictions regarding the COVID-19 vaccination were included. Eight knowledge-based questions (marked as K for Knowledge, K1-K8: “Yes = 1” vs. “No = 0,” score range: 0 to 8), designed to measure participants’ knowledge score about the COVID-19 vaccine, were included. The participants’ scores indicated how well-versed they were [18]. Also, participants were questioned about their sources of information about COVID-19 vaccine.

Then, the participants were divided into three groups according to the answer to COVID-19 vaccine intention question. The group who answered “Yes, absolutely” or “Yes, probably” was considered as vaccine acceptant group (VA). Those who answered “No, probably not” or “I do not know” were considered as vaccine hesitant group (VH). Participants were considered as vaccine resistant (VR) when their answers were “No, certainly not” or “No, probably not”. Questions about COVID-19 barriers and motivators were also included.

#### Status of COVID-19 vaccination

Finally, COVID-19 vaccination status was questioned, and vaccinated participants were asked about the received vaccine type and side effects including allergy, fever, rash, rigors, bone aches, fatigue, headache, GIT upset and chest symptoms.

## Statistical analysis

The responses of participants were documented and conveyed to excel spread sheets. Statistical Package for Social Science (SPSS) version 22 was used to analyze the gathered data. Quantitative data were presented as means with standard deviation (SD) for parametric variables or medians (min-max) for nonparametric variables, and qualitative data were given as numbers and percentages. Shapiro-Wilk test was employed to evaluate the normality of the distribution of variables. One-way ANOVA test was used for parametric variables to compare between the study groups, whereas Kruskal-Wallis test was utilized for non-parametric variables. Comparing qualitative variables was done using the Chi-square test. Significant was defined as a *P* value of less than 0.05.

## Results

This study was conducted on 455 HCWs (response rate,75.8%), their mean age was 36.55 years (SD = 10.31). More than 80% of the participants were females. About half of them (50.1%) were from rural origin. Fifty-two were hypertensive (11.4%) and 44 were diabetic (9.7%). Other sociodemographic and clinical data of the participants are illustrated in Table 1.

**Table 1:** Sociodemographic and clinical data of the study health care workers according to their intention to get COVID-19 vaccine (*n* = 455)

Variable n (%), mean ± SD	Total (*n* = 455)	Vaccine Acceptant group (*n* = 321)	Vaccine Hesitant group (*n* = 80)	Vaccine Resistsnt group (*n* = 54)	P
*Gender*					
Female	367 (80.7)	254 (79.1)	68 (85)	45 (83.3)	0.428
Male	88 (19.3)	67 (20.9)	12 (15)	9 (16.7)	
*Age, years*	36.55 ± 10.31	35.92 ± 10.21	37.09 ± 10.19	39.46 ± 10.66	0.057
18–24	51 (11.2)	40 (12.5)	7 (8.8)	4 (7.4)	0.539
25–35	182 (40)	134 (41.7)	32 (40)	16 (29.6)	
36–45	132 (29)	88 (27.4)	25 (31.3)	19 (35.2)	
46–60	86 (18.9)	57 (17.8)	15 (18.8)	14 (25.9)	
more than 60	4 (0.9)	2 (0.6)	1 (1.3)	1 (1.9)	
*Marital status*					
Single/divorced/widowed	128 (28.1)	103 (32.1)	19 (23.8)	6 (11.1)	0.004*
Married	327 (71.9)	218 (67.9)	61 (76.3)	48 (88.9)	
*Residence*					
Rural	228 (50.1)	148 (46.1)	53 (66.3)	27 (50)	0.006*
Urban	227 (49.9)	173 (53.9)	27 (33.8)	27 (50)	
*Smoking*					
Never	421 (92.5)	296 (92.2)	76 (95)	49 (90.7)	0.687
Former smoker	8 (1.8)	5 (1.6)	2 (2.5)	1 (1.9)	
Current smoker	26 (5.7)	20 (6.2)	2 (2.5)	4 (7.4)	
Active lifestyle	360 (79.1)	257 (80.1)	57 (71.3)	46 (85.2)	0.112
*Socioeconomic status*					
Low	44 (9.7)	26 (8.1)	10 (12.5)	8 (14.8)	0.490
Average	381 (83.7)	274 (85.4)	65 (81.3)	42 (77.8)	
High	30 (6.6)	21 (6.5)	5 (6.3)	4 (7.4)	
*Comorbidities*					
Diabetes	44 (9.7)	31 (9.7)	8 (10)	5 (9.3)	0.990
Hypertension	52 (11.4)	38 (11.8)	7 (8.8)	7 (13)	0.689
Chronic respiratory disease	9 (2)	6 (1.9)	2 (2.5)	1 (1.9)	0.934
Psychiatric disorder	7 (1.5)	3 (0.9)	3 (3.8)	1 (1.9)	0.184
Ischemic heart disease	5 (1.1)	3 (0.9)	0	2 (3.7)	0.114
Autoimmune Disease	4 (0.9)	3 (0.9)	1 (1.3)	0	0.735
Chronic renal disease	3 (0.7)	1 (0.3)	2 (2.5)	0	0.078
Chronic liver disease	5 (1.1)	2 (0.6)	2 (2.5)	1 (1.9)	0.302
Hypersensitivity	67 (14.7)	47 (14.6)	11 (13.8)	9 (16.7)	0.894
Obesity	29 (6.4)	21 (6.5)	5 (6.3)	3 (5.6)	0.962
Others	20 (4.4)	12 (3.7)	3 (3.8)	5 (9.3)	0.178

*P < 0.05

**Fig. 1:**
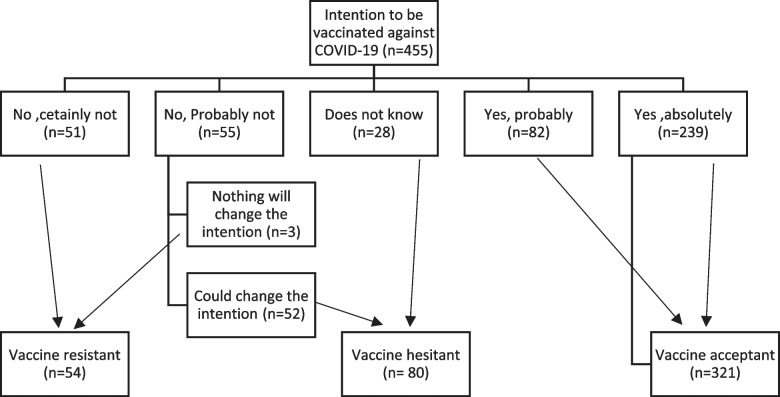
Classification of the study health care workers according to intention to receive COVID-19 vaccine (*n* = 455)

Participants were classified according to intention to receive COVID-19 vaccine into 3 groups: the largest group was VA group (321,70.5%), followed by VH (80,17.6%) then VR group (54,11.9%) (Fig. 1).

Occupational data are shown in Table 2. The participants included nurses (37.8%), physicians (25.9%), administrators (10.1%), workers or security officers (5.3%), radiology or laboratory technicians (4.4%), pharmacists (2.2%) and dentist (1.3%). About one third (31.6%) were working in outpatient, radiology, and hemodialysis units while 27.5% were working in inpatient wards, ambulance, emergency, operation and delivery rooms. Most of the participants (80%) were working in patient-facing areas. The frequency of contact with COVID-19 patients in the workplace was reported to be daily by 211 participants (46.4%), weekly by 83 (18.2) and monthly by 65 (14.3%).

**Table 2:** Occupational data of the study health care workers (*n* = 455)

Variable	Total (*n* = 455) n (%)	Vaccine Acceptant group (*n* = 321) n (%)	Vaccine Hesitant group (*n* = 80) n (%)	Vaccine Resistsnt group (*n* = 54) n (%)	P
*Occupation*					
Physician	118 (25.9)	102 (31.8)	13 (16.3)	3 (5.6)	< 0.001*
Nurse	172 (37.8)	105 (32.7)	39 (48.8)	28 (51.9)	
Dentist	6 (1.3)	6 (1.9)	0	0	
Pharmacist	10 (2.2)	6 (1.9)	3 (3.8)	1 (1.9)	
Administrator	46 (10.1)	38 (11.8)	4 (5)	4 (7.4)	
Radiology or laboratory technician	20 (4.4)	16 (5.)	3 (3.8)	1 (1.9)	
A worker or security officer	24 (5.3)	10 (3.1)	9 (11.3)	5 (9.3)	
others	59 (13)	38 (11.8)	9 (11.3)	12 (22.2)	
*Occupation settings*					
Offices	45 (9.9)	32 (10)	11 (13.8)	2 (3.7)	0.012*
Labs	29 (6.4)	28 (8.7)	0	1 (1.9)	
Patient facing non-clinical (community or hospital pharmacy)	12 (2.6)	7 (2.2)	4 (5)	1 (1.9)	
Outpatient, radiology, GP, hemodialysis unit	144 (31.6)	99 (30.8)	29 (36.3)	16 (29.6)	
Inpatient wards, ambulance, ER, Operation room, delivery room	125 (27.5)	91 (28.3)	21 (26.3)	13 (24.1)	
Intensive care	22 (4.8)	16 (5)	3 (3.8)	3 (5.6)	
Others	78 (17.1)	48 (15)	12 (15)	18 (33.3)	
Contact with patients or working in patient-facing areas	364 (80)	258 (80.4)	63 (78.8)	43 (79.6)	0.945
*Frequency of contact with COVID-19 patients in the workplace*					
Never	96 (21.1)	66 (20.6)	17 (21.3)	13 (24.1)	0.272
Daily	211 (46.4)	150 (46.7)	33 (41.3)	28 (51.9)	
Weekly	83 (18.2)	62 (19.3)	12 (15)	9 (16.7)	
Monthly	65 (14.3)	43 (13.4)	18 (22.5)	4 (7.4)	

*P < 0.05

As shown in Table 3, about two thirds (63.3%) of the participants reported *SARS*-*CoV*-*2* infection among their household or close friends. Additionally, 30.8% and 21.5% of our cohort, respectively, reported relative hospitalization or mortality due to *SARS*-*CoV*-*2* infection. About one third (33.4%) of the subjects had previously contracted COVID-19, with symptoms lasting an average of 6 days. In terms of the COVID-19 course in previously infected individuals, 32.2% had a mild infection that did not require hospitalization or interfere with daily activities, 63.2% had a more complicated disease in the form of a prolonged disease course that did interfere with daily activities, and 4.6% had severe symptoms necessitating hospitalization. There was no ICU admission reported by any of the participants.

**Table 3:** Clinical data of SARS-CoV-2 infection reported by the study health care workers (n = 455)

Variable n (%), median (min-max)	Total (*n* = 455)	Vaccine Acceptant group (*n* = 321)	Vaccine Hesitant group (*n* = 80)	Vaccine Hesitant group (*n* = 54)	P
COVID-19 diagnosis among the household or close friends	288 (63.3)	208 (64.8)	45 (56.3)	35 (64.8)	0.978
A relative has been hospitalized because of SARS-CoV-2 infection	140 (30.8)	105 (32.7)	23 (28.8)	12 (22.2)	0.276
A relative died from SARS-CoV-2 infection	98 (21.5)	74 (23.1)	13 (16.3)	11 (20.4)	0.406
Infected with COVID-19	152 (33.4)	108 (33.6)	25 (31.3)	19 (35.2)	0.751
Duration of symptoms, from the first day you became ill until symptoms resolved, days	6 (1–45)	6 (1–45)	1 (1–21)	12 (1–45)	0.002*
*Course of SARS-CoV-2 infection*					
Not hospitalized and no difficulties in performing daily activities	49/152 (32.2)	39/108 (36.1)	5/25 (20)	5/19 (26.3)	0.029*
Not hospitalized but had some difficulties in performing my daily activities	96/152 (63.2)	67/108 (62)	16/25 (64)	13/19 (68.4)	
Hospitalized and did not require ICU	7/152 (4.6)	2/108 (1.9)	4/25 (16)	1/19 (5.3)	
Hospitalized and required ICU care	0	0	0	0	

*P < 0.05

Most participants believed that they had a higher risk of contracting COVID-19 (71.6%). More than 64% thought that they were at risk for vaccination side effects, and the percentage of this perception was significantly higher in VR group (88.9%). Regarding perceptions of efficacy, security, utility, and estimated knowledge of conventional vaccines, there was a statistically significant difference between the VA, VH, and VR groups. Additionally, the knowledge score in the VA group was significantly higher than other groups (*p* = 0.001). Other beliefs and attitudes towards COVID-19 vaccination are illustrated in Table 4.

**Table 4:** Perception, beliefs and attitudes of the study health care workers towards SARS-CoV-2 infection, conventional and COVID-19 vaccination (*n* = 455)

Statement n (%), median (min-max)	Total (*n* = 455)	Vaccine Acceptant group (*n* = 321)	Vaccine Hesitant group (*n* = 80)	Vaccine Hesitant group (*n* = 54)	P
*SARS-CoV-2 infection*					
Do you think that you at higher risk of contracting COVID-19?	326 (71.6)	230 (71.7)	54 (67.5)	42 (77.8)	0.485
Do you think that you may have more severe COVID-19 due to chronic illness?	261 (57.4)	182 (56.7)	44 (55)	35 (64.8)	0.469
Do you think that you are at higher risk of COVID-19 vaccine adverse events	295 (64.8)	193 (60.1)	54 (67.5)	48 (88.9)	0.000*
Self-rated knowledge level about COVID-19					
Very bad	18 (4)	11 (3.4)	5 (6.3)	2 (3.7)	0.011*
Bad	35 (7.7)	15 (4.7)	13 (16.3)	7 (13)	
Average	220 (48.4)	156 (48.6)	40 (50)	24 (44.4)	
Good	132 (29)	100 (31.2)	15 (18.8)	17 (31.5)	
Very good	50 (11)	39 (12.1)	7 (8.8)	4 (7.4)	
*Conventional vaccination (excluding COVID-19 vaccines)*					
Efficacy	6 (0–10)	6 (0–10)	5 (0–10)	5 (0–10)	0.001*
Security	6 (0–10)	7 (0–10)	5 (0–10)	3 (0–10)	0.000*
Usefulness	8 (0–10)	8 (0–10)	5 (0–10)	5 (0–10)	0.000*
Estimated knowledge	7 (0–10)	7 (0–10)	5 (0–10)	5 (0–10)	0.001*
Knowledge score	4 (0–8)	4 (0–8)	3 (0–6)	3 (0–8)	< 0.001*
*COVID-19 vaccination*					
How important do you perceive the COVID-19 vaccine to be?	354 (77.8)	284 (88.5)	43 (53.8)	27 (50)	< 0.001*
How important you think that everyone in the community should get the COVID-19 vaccine once available?	329 (72.3)	274 (85.4)	33 (41.3)	22 (40.7)	< 0.001*
Vaccination of COVID-19 should always be compulsory once it is available	247 (54.3)	211 (65.7)	26 (32.5)	10 (18.5)	< 0.001*
Do you have concerns regarding the COVID-19 vaccination?	312 (68.6)	205 (63.9)	59 (73.8)	48 (88.9)	< 0.001*
Vaccination of COVID-19 should always be compulsory for health care workers once it is available	311 (68.4)	256 (79.8)	37 (46.3)	18 (33.3)	< 0.001*
I think that approval of the vaccine guarantees its safety	204 (44.8)	173 (53.9)	20 (25)	11 (20.4)	< 0.001*
Do you have concerns regarding the adverse effects of the vaccine	316 (69.5)	211 (65.7)	60 (75)	45 (83.3)	.017*
Do you have concerns about the ineffectiveness of the vaccine	106 (23.3)	77 (24)	18 (22.5)	11 (20.4)	.830
Having a prior bad experience with any vaccines and their adverse reactions	72 (15.8)	34 (10.6)	20 (25)	18 (33.3)	< 0.001*
Do you have concerns for the acquisition of COVID-19 from the vaccine	248 (54.5)	148 (46.1)	54 (67.5)	46 (85.2)	< 0.001*
Do you think that COVID-19 vaccination is the best protective method against COVID-19	261 (57.4)	223 (69.5)	23 (28.8)	15 (27.8)	< 0.001*

*P < 0.05

**Fig. 2:**
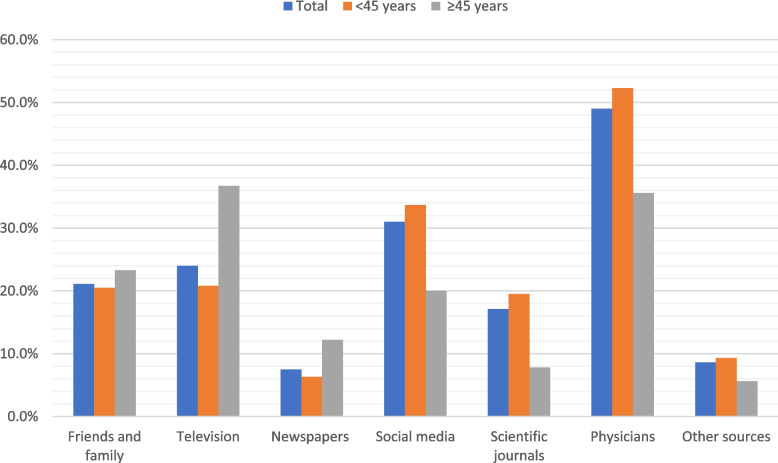
Sources of information about COVID-19 vaccine among the study health care workers according to the age (*n* = 455)

The sources of information about the COVID-19 vaccination are displayed in Fig. 2. Physicians were reported to be primary source of COVID-19 vaccine information in about half of the participants (49%) followed by social media for young participants (< 45 years) and television for others (≥45 years).

**Fig. 3:**
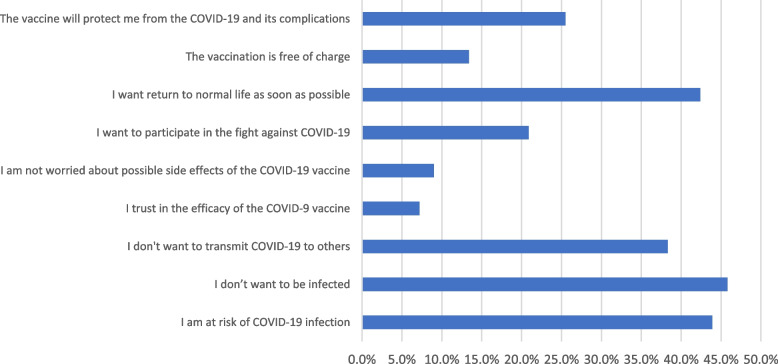
The motivators of COVID-19 vaccination among COVID-19 vaccine acceptant group (*n* = 321)

**Fig. 4:**
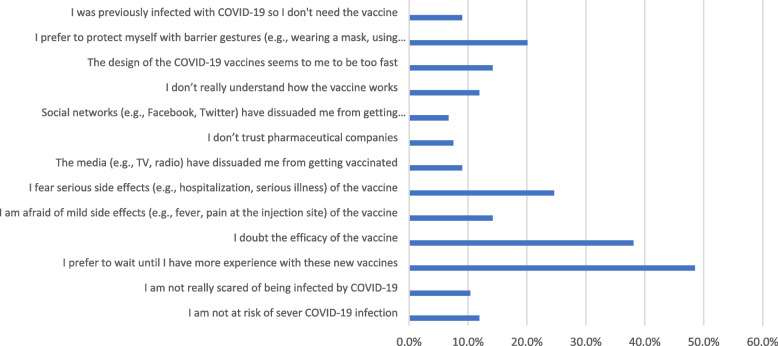
Barriers of COVID-19 vaccination among COVID-19 vaccine hesitant and resistant groups (*n* = 134)

**Fig. 5:**
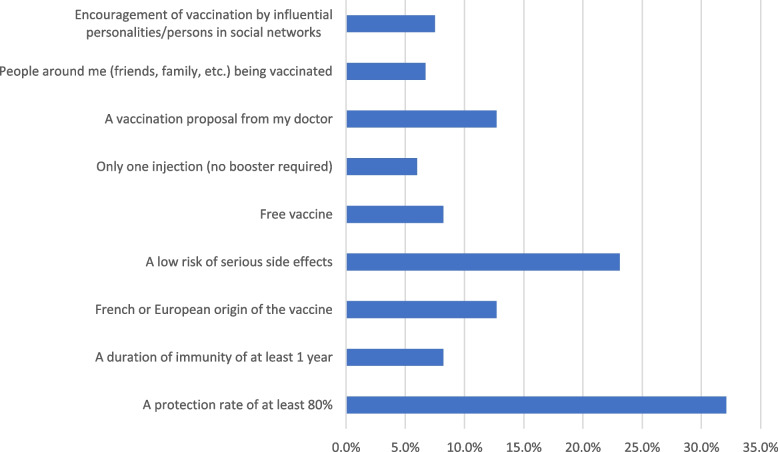
Opportunities to change decision regarding COVID-19 vaccination among COVID-19 vaccine hesitant and resistant groups (*n* = 134)

Fear of infection, being at high risk of infection, and the desire to resume normal life were the main drivers for COVID-19 vaccination in the VA group as shown in Fig. 3. On the other hand, the major barriers to the COVID-19 vaccination in the VR group were waiting for additional experience with these new vaccines and having doubts about the vaccines’ efficacy as illustrated in Fig. 4. The most significant factors that could influence the decision of the VH group are illustrated in Fig. 5.

Among participants, 179 (39.3%) had received COVID-19 vaccine. Astrzeneca (53.1%) and Sinopharm (20.7%) were the two most frequently received vaccines. The two most frequently reported vaccination adverse effects were fever (45.3%) and body aches (55.3%). However, as demonstrated in Table 5, the least common side effects were rash (3.4%) and anaphylaxis (1.1%).

**Table 5:** List of types and adverse effects of COVID-19 vaccines in the study health care workers who have received COVID-19 vaccine (*n* = 179,39.3%)

Variables	Vaccinated HCW (*n* = 179) n (%)
*Type of COVID-19 vaccine received*	
Astrazeneca	95 (53.1)
Sinopharm	37 (20.7)
Sinovac	28 (15.6)
Pfizer	14 (7.8)
Sputnik	4 (2.2)
Johnson & Johnson’s Janssen Covid-19 Vaccine	1 (0.6)
*Adverse effects of COVID-19 vaccines*	
Widespread muscle/joint pain	99 (55.3)
Fever or chills	81 (45.3)
Headache	77 (43)
Local skin reaction	66 (36.9)
Fatigue or sleepiness	49 (27.4)
Chest pain – palpitations	21 (11.7)
Nausea	14 (7.8)
Poor appetite	13 (7.3)
Vomiting	8 (4.5)
Rash	6 (3.4)
Anaphylaxis	2 (1.1)

## Discussion

The COVID-19 vaccination is one of the most crucial strategies for containing the COVID-19 pandemic. HCWs are more likely to contract COVID-19 than the general population. So, their attitude toward the vaccination is crucial since it can determine how well the general population responds to it [19].

The aim of this study was to assess Egyptian HCWs’ attitudes toward the COVID-19 vaccine. The 455 HCWs who participated in this study were divided into three groups—VA, VH, and VR—based on their attitude toward COVID-19 vaccine. Most of participants were vaccine accepting (70.5%). Of the participants, one-third had previously contracted COVID-19, and the majority of them had prolonged disease course. The majority of participants believed that they were at high risk for both *SARS*-*CoV*-*2* infection and vaccination side effects. There was a statistically significant difference between the VA, VH, and VR groups regarding perception and knowledge of conventional and COVID-19 vaccines. Fear of infection and the desire to resume normal life were the main drivers behind the COVID-19 vaccination. The two major barriers, however, were waiting for additional expertise and having doubts about the effectiveness of the vaccines.

About one third (33.4%) of participants reported having previously contracted COVID-19, a rate that was comparable to that in a prior study also involving Egyptian HCWs [20]. Other studies done in nations with higher incomes found a much lower percentage of *SARS*-*CoV*-*2* infection [21, 22]. In a different study on the general population, the percentage of people with a history of prior *SARS*-*CoV*-*2* infection was around 20%, and there was no difference in this percentage between those in the medical and non-medical fields [23].

Most participants in this study were vaccine accepting (70.5%). This high rate of vaccine acceptance was comparable to the findings of earlier studies conducted on family physicians [24], primary care physicians [25], pediatricians [26], pharmacists [27], dentists [28], medical students [29] and HCWs [30, 31]. Additionally, a recent meta-analysis of ten studies on dental practitioners and students indicated that vaccine acceptability was higher in middle eastern nations [32].

The majority of individuals in other studies, however, were shown to be hesitant to receive COVID-19 vaccine [33, 34]. In a global study that was conducted in 12 countries, the majority of participants were in favor of vaccinations; nevertheless, Egypt and African nations had the lowest vaccination acceptance rates. Higher income countries showed greater vaccine adoption in the same study [35]. In another survey of nurses and midwives, the VR group was found to have the highest percentage (more than 90%), with participants worried about the vaccine’s side effects and how rapidly development occurred [36]. Notably, vaccine acceptance in recent surveys was higher than earlier one. This may be ascribed to more recent and available studies, public vaccination campaigns, and political motivations.

Participants from urban areas were more vaccine accepting and these results were consistent with the finding reported by Biswas et al., in which, HCWs working at urban areas, were more vaccine accepting [37]. Rural communities may have limited access to health care services, which may contribute to the gap in vaccine acceptance. As a result, specific initiatives are required to boost vaccine confidence and bridge the gap between urban and rural communities. Public health practitioners could focus on engaging with community-based organisations to increase vaccine confidence, guarantee equitable vaccine access, and urge rural residents to stay up to date on necessary COVID-19 vaccines.

There was no difference as regard age, gender or working with COVID-19 patients in our cohort. In previous studies, it was observed that males and physicians were more accepting of vaccinations than females and nurses [22, 38, 39]. Because of this, physicians play a significant part in increasing public acceptability of the COVID-19 vaccine.

Previous exposure to *SARS*-*CoV*-*2* infection is associated with higher vaccine acceptance [40]. However, there was no difference between VA, VH, VR groups in this study with relation to prior or family history of *SARS*-*CoV*-*2* infection.

More acceptance of the vaccine was linked to stronger awareness of the COVID-19 vaccine and higher knowledge scores [34]. In the present study, there was a significant difference between the 3 groups as regard perception towards conventional and COVID-19 vaccination and knowledge score. These results were in line with previous studies, which showed a substantial difference between the VR, VH, and VA groups in terms of attitudes toward vaccination and perceptions of the safety of the COVID-19 vaccine [33, 41].

Physicians were reported to be the primary source of information in about half of our participants (49%) followed by social media for young participants (< 45 years) and television for others (≥45 years). It was found that higher education HCWs rely mainly on institutional sources and scientific literature. However, lower education HCWs rely on internet, mass media and opinions of family and friends [39]. Social and mass media are important sources of information [42]. However, using social media as a source of information is associated with more vaccine hesitancy [43], while using national websites is associated with less hesitancy [33].

In this study, fear of infection, being at high risk of infection, and the desire to resume normal life were the main drivers for COVID-19 vaccination in the VA group. Similar causes were reported by previous studies conducted on family physicians and other HCWs [24, 30].

Concerns about safety and efficacy are also important barriers against vaccination [19, 29]. In this study, the major barriers to the COVID-19 vaccination in the VR group were waiting for additional experience with these new vaccines and having doubts about the vaccines’ efficacy. It was previously reported that the biggest obstacles to vaccination acceptability were the quick creation of the vaccine and a lack of adequate information [42, 44]. Additionally, a lack of clinical trials and concern about side effects are the main reasons why people are hesitant to obtain the vaccination. Providing this group with adequate factual information will boost their acceptance of the vaccine [20].

Among participants, 179 (39.3%) had received COVID-19 vaccine. In certain studies, a lower vaccination rate was noted [19]. However, some research indicated a far greater rate [45, 46]. Astrzeneca (53.1%) and Sinopharm (20.7%) were the two most frequently received vaccines in our cohort. This was according to which was available for each participant. In general, m RNA vaccines are the most preferred vaccines [46]. Additionally, Pfizer and Astrazeneka vaccines are the most popular vaccination types in Arabic-speaking and African countries [47, 48].

Furthermore, there was a disparity between vaccination acceptants (70%) and vaccine recipients (40%) among our cohort. This is due to a limited supply of COVID-19 vaccine at the time of the trial, and the government devised a policy for the sequential inclusion of high priority groups, including HCWs. As a result, all HCWs who accepted to vaccination received it in sequence.

In conclusion, our results emphasize the value of including HCWs in pandemic vaccination campaigns. HCWs were very accepting of COVID-19 vaccines and played a crucial role in assisting patients in their vaccine decisions despite having expressed vaccine concerns. The community adopts these perceptions because of exposure to false information, which is magnified by the media. Recognizing and addressing issues at all levels is essential for increasing the reach of COVID-19 vaccination campaigns. We recommend that this study be replicated using a qualitative research approach to bridge the gap identified between practice and attitude. This study has many strengths. First, we performed a multicenter study including HCWs with various levels of education and employment experiences. Second, this study sheds essential light on the potential obstacles to and drivers behind vaccination among HCWs who are an important source of human resources in vaccination. Third, this study offers important information regarding the actual conversion of vaccine acceptance into vaccine uptake as well as adverse reactions following vaccination.

However, the study has several limitations. First, because the study was cross-sectional, it was challenging to evaluate the causes and effect relationships. Second, we employed convenience sampling, which could have biased the results; those who were accepting the COVID-19 vaccine may be more likely to participate in the survey and this could explain the difference in the results with other studies that reported less acceptance, and a different preference concerning sex. Third, some sites collected data before vaccination began, while others did so after it had begun, which may have an impact on HCWs’ attitudes. Hence, as more information about the safety and efficacy of COVID-19 vaccines becomes available, individuals may have different attitudes towards vaccination.

## Data Availability

The datasets generated during and/or analysed during the current study are available from the corresponding author on reasonable request.

## Abbreviations

CAPMAS: Central Agency for Public Mobilization and Statistics
COVID-19: Coronavirus disease 2019
HCWs: Healthcare workers
SARS-CoV-2: Severe acute respiratory syndrome coronavirus 2
SPSS: Statistical Package for Social Science
WHO: World Health Organization
